# More than density: validating a mammographic masking prediction model in Dutch breast cancer screening

**DOI:** 10.1007/s00330-025-11687-x

**Published:** 2025-05-29

**Authors:** Sarah D. Verboom, James G. Mainprize, Jim Peters, Mireille Broeders, Martin J. Yaffe, Ioannis Sechopoulos

**Affiliations:** 1 Department of Medical Imaging, Radboud University Medical Center, Nijmegen, The Netherlands; 2 Dutch Expert Centre for Screening (LRCB), Nijmegen, The Netherlands; 3 Physical Sciences, Sunnybrook Research Institute, Toronto, Canada; 4 IQ Health Science Department, Radboud University Medical Center, Nijmegen, The Netherlands; 5 Ontario Institute for Cancer Research, Toronto, Canada; 6 Technical Medicine Center, University of Twente, Enschede, The Netherlands

**Keywords:** Breast neoplasms, Mammography, Breast density, Perceptual masking, Early detection of cancer

## Abstract

**Objectives:**

To validate a lesion masking prediction model, Mammatus, previously developed on a North American cohort, on a larger retrospective breast cancer screening cohort from a single center in the Netherlands.

**Materials and methods:**

Mammatus was applied to all digital mammography screening examinations with a unilateral invasive breast cancer that was either diagnosed at screening or within 24 months after a negative screening, called interval cancers. All mammograms were retrospectively evaluated for the visibility of malignant masses using all available imaging and clinical information.

The area under the receiver operator characteristic (ROC) curve (AUC) when using Mammatus to distinguish examinations with screen-detected cancers (assumed low masking risk) from interval cancers (assumed high masking risk) was computed. The AUC was compared to that of the original cohort and to that obtained using volumetric breast density (VBD) as a predictor. A second tghree-category ROC analysis was performed, with interval cancers that were retrospectively visible classified as intermediate lesion masking.

**Results:**

Mammatus achieved an AUC of 0.69 (95% CI: 0.66–0.73) for distinguishing between screen-detected-cancer exams (*n* = 635) and interval-cancer exams (*n* = 304). This performance did not differ from the original study (AUC = 0.75 (95% CI: 0.68–0.82), *p* = 0.15), and outperformed VBD (AUC = 0.66 (95% CI: 0.63–0.70, *p* = 0.019). Mammatus was better at identifying mammograms at low risk of lesion masking (AUC = 0.73 (95% CI: 0.70–0.76)) compared to those with high risk (AUC = 0.69 (95% CI: 0.64–0.74)).

**Conclusion:**

Mammatus performed well in predicting breast cancer-masking risk in a Dutch screening cohort. This suggests that adding information other than density facilitates the prediction of lesion masking.

**Key Points:**

***Question*** *Mammographic lesion masking prediction models, such as Mammatus, require external validation in other screening programs before clinical application is possible*.

***Findings*** *Mammatus maintained similar performance in predicting lesion masking in a Dutch screening cohort and showed added benefit compared to VBD*.

***Clinical relevance*** *An externally validated lesion masking prediction model for digital mammography could potentially be used to identify screened women who could benefit from supplemental or alternative screening, with better accuracy than VBD alone*.

**Graphical Abstract:**

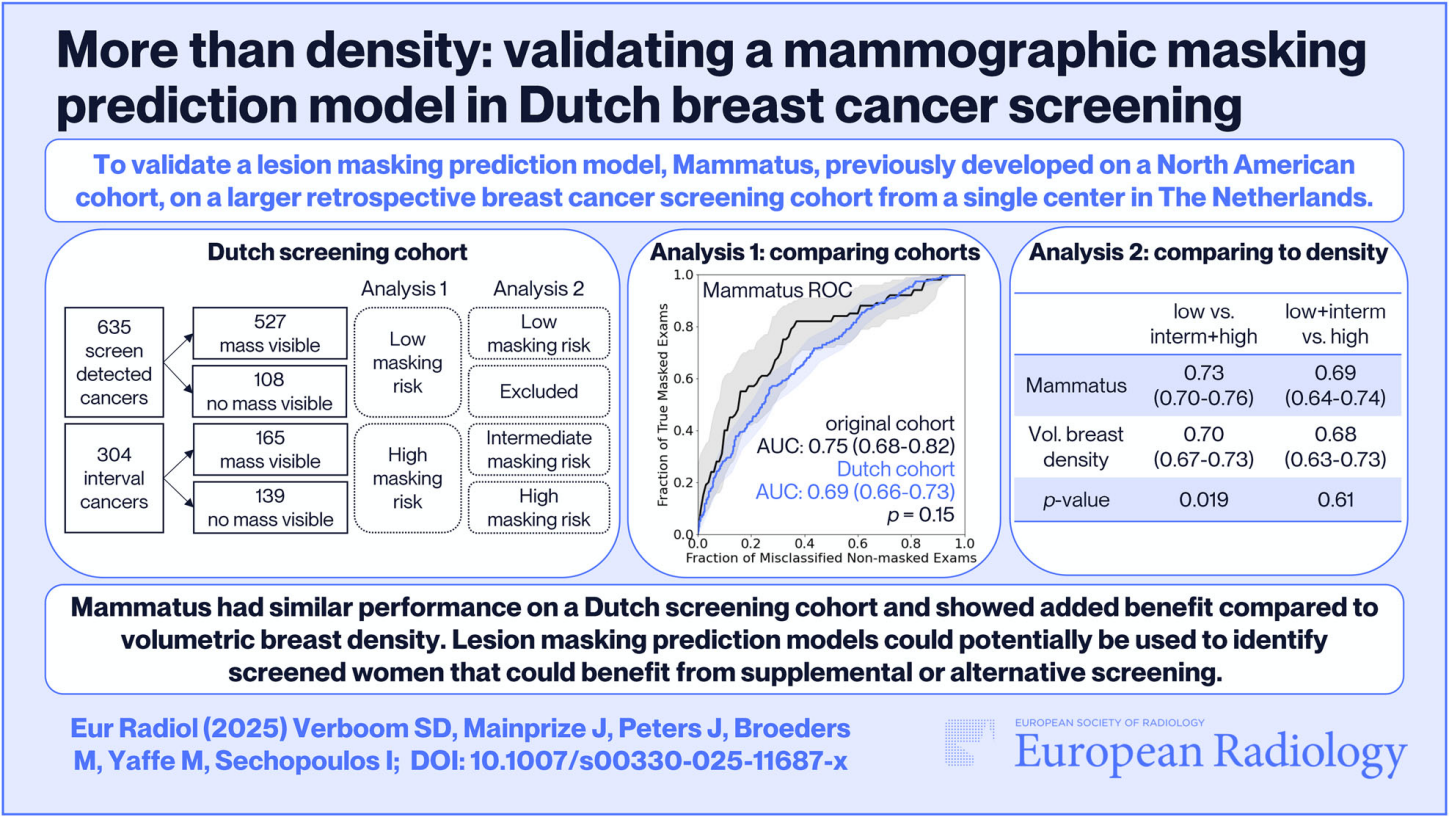

## Introduction

Digital mammography is the standard modality employed in most breast cancer screening programs. In the Netherlands, women between the ages of 50 and 75 are invited for biennial screening. Despite the high cancer detection rate of 6.9 per 1000 [1], there are still 2.2 cancers per 1000 women screened that are diagnosed by other means before the next screening round, referred to as interval cancers. Masking of cancer lesions due to high breast density is one of the known factors responsible for interval cancers [2]. Masking results from the superposition of fibroglandular tissue in the image can cause a lesion to be obscured. Although high volumetric breast density (VBD) increases the risk of mammographic masking, it is not the sole predictor of lesion masking risk [3, 4]. Predicting lesion masking risk could identify cases where extra attention is required by the radiologists during reading or could identify candidates for supplemental imaging, using a modality whose sensitivity is not reduced in the presence of dense breast tissue. These may include MRI, ultrasound, contrast-enhanced mammography, or breast CT.

There have been several published attempts to estimate lesion masking risk [5–7]. One previously developed algorithm, Mammatus (Sunnybrook Research Institute, Canada), uses radiomics features from density and detectability maps created by a model observer, in addition to clinical factors such as age and body mass index, to estimate the risk of lesion masking [5]. This algorithm is an investigational tool only and has not been approved for clinical use. It has been validated using screening mammograms from both screen-detected cancers, where the lesion was not masked, and interval cancers, where the lesion was likely masked. The latter group relies on the assumption that the cancers were missed at screening and diagnosed outside of the screening program due to masking, but there are other possible reasons. For example, the lesion can fall outside the field of view, be overlooked, or be misinterpreted as benign. It is also possible that the cancer was fast-growing and developed between screening rounds. Furthermore, the model was tested only on a limited dataset comprising 147 screen-detected cancers and 67 interval cancers.

The objective of this study is to test the Mammatus lesion masking prediction model on a cohort from the Dutch Breast Cancer Screening Program with retrospective verification of lesion masking.

## Materials and methods

### Dataset

The lesion masking risk estimation model was tested on retrospectively collected digital mammograms acquired at a single screening center in the Netherlands between 2008 and 2018, all from Lorad Selenia mammography systems (Hologic). In the Netherlands, all women between the ages of 50 and 75 are invited biennially for mammographic screening. Women who participate in the screening program are informed that their data can be used for scientific purposes. Their data was not used if women chose to opt out of data provision. The Radboudumc ethics committee declared that this study falls outside the scope of the Dutch Medical Research involving Human Subjects Act and could be carried out without approval of an Institutional Review Board. In the cohort, there were 2610 cancer diagnoses, of which 1916 were diagnosed as a result of the screening examination and 694 were diagnosed clinically, called interval cancer. Only unilateral, and invasive cancers were included; in situ, micro-invasive, and metachronous cancers were excluded.

### Reference standard

**Fig. 1:**
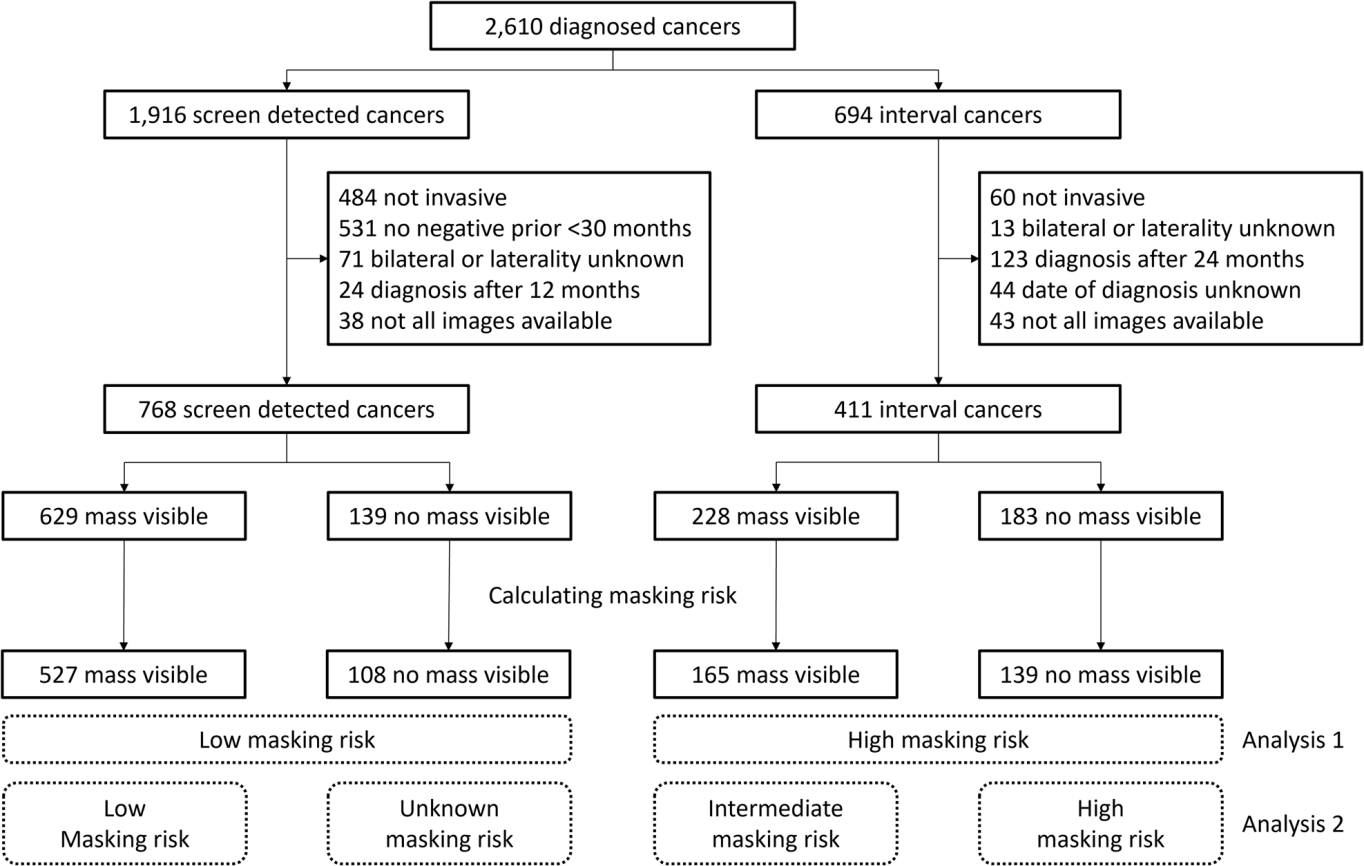
Flowchart of the inclusion of mammographic screening examinations

Two groups of digital mammograms were included, the first consisted of positive screening examinations with screen-detected cancers, while the second group consisted of mammograms from the last negative screening round before an interval cancer was diagnosed within 24 months. Cancers were considered as having been screen-detected when diagnosis occurred within 12 months after the positive screening, and there was a negative prior screening less than 30 months before the positive screening. Positive screenings without a known negative prior were excluded to avoid large or late-stage cancers that may have been detectable in mammograms with either high or low masking risk. Therefore, first-round screenings were also excluded. Mainprize et al [5] tentatively labeled these two groups as being associated with low and high masking risk, respectively. This definition was also used for analysis 1 (Fig. 1) to provide an accurate comparison. There are two main limitations in this definition of masking risk. First, lesion masking has a negative effect on the detection of masses, but less so on the detection of other abnormalities, such as calcifications [2]. Second, not all interval cancers are missed due to masking; there are many other possible causes for cancers not being screen-detected, including reader error or the lesion being outside of the field of view. To partially correct for these limitations, all included examinations were retrospectively analyzed for a visible mass at the cancer location. This analysis was performed as part of another study [8] in which researchers, supervised by an experienced breast radiologist, retrospectively identified the masses corresponding to the invasive breast cancers and manually drew the lesion contour. For this task, the researchers had information on the localization of the lesion, available through linkage with the Netherlands Cancer Registry, and the output of a state-of-the-art detection algorithm during the procedure, which performs on par with experienced radiologists [9].

Therefore, after retrospective analysis, all mammographic examinations were classified as one of four different categories for analysis 2 (Fig. 1): (1) examinations with screen-detected cancer visible as a mass, (2) examinations with screen-detected cancer without a visible mass, (3) last negative examination before an interval cancer with a visible mass, and (4) last negative examination before an interval cancer without a visible mass. In the first category, the lesion was not masked, and therefore, it was considered that the masking risk was low. In the second category, the cancer was also detected and therefore not masked. However, the cancer did not appear as a mass (but for example as an architectural distortion, asymmetry, or calcification), and therefore, it is unknown what the lesion masking risk was in this examination. In the third category, the cancer was initially missed, but was visible as a mass in retrospect. This category can be classified as having an intermediate masking risk, making the lesion difficult to interpret or easier to miss. In the last category, the cancer was missed, and no mass was visible in retrospect. Therefore, this category most likely involves high lesion masking risk. However, this category can also include true interval cancers that were not present during the screening examination.

### Lesion masking risk prediction

The lesion masking risk prediction model, Mammatus, introduced by Mainprize et al [5] is based on a combination of features from a synthetic lesion detectability map, a volumetric density map based on an in-house algorithm, and clinical features. The detectability maps are generated by calculating the detection signal-to-noise ratio for a lesion object of Gaussian profile against the breast background texture at a series of locations on the mammogram of each subject. From both the detectability and density maps, several radiomic features are calculated that are predictive of masking risk. The most important features are selected with a stepwise variable selection method that uses the Akaike Information Criterion. The final model is based on the standard deviation of the detectability map, the correlation of the gray-level co-occurrence matrix of the density map, and age.

Mammatus uses a self-calibration routine where it extracts an open beam signal from the background of unsaturated images. This signal is dependent on the tube voltage, anode/filter combination, the age of the tube, and signal response/gain calibration of the detector. If it is not possible to get a reference value of the open beam signal, signal calibration is not available for that image, and therefore, the mammogram cannot be processed. Future development on Mammatus will include more robust methods for extrapolating the open beam signal to fill in missing values for these combinations of technique factors.

It is known that the lesion masking risk is influenced by the volumetric density of the breast. To evaluate the added value of the lesion masking risk prediction model compared to VBD, the VBD for each examination was estimated using commercial software (version 3.5.0, Volpara Health Technologies).

### Model evaluation

Mammatus and Volpara were applied to all available images, and the outputs were averaged over the medio-lateral oblique and cranio-caudal views of both sides. Two receiver operator characteristic (ROC) analyses were performed for the lesion masking score of Mammatus and for VBD. The first analysis was designed to closely match the methods of the original paper and therefore provide a fair comparison. The second analysis used the improved ground truth based on the retrospective analysis.

The first ROC analysis evaluates the discriminative property of Mammatus and VBD between mammograms of screen-detected cancers with assumed low masking risk (categories 1 and 2) and mammograms before interval cancers with assumed high masking risk (categories 3 and 4). This analysis is similar to that of the original work by Mainprize et al [5] and allows comparison of the area under the receiver operator characteristic curve (AUC) to that reported there with a *Z*-test. The AUC of Mammatus was also compared to the AUC of VBD as a predictor with a single-reader multi-case analysis (MRMCaov for R, version 0.3.0 [10, 11]) and a jackknife function to estimate performance metric covariances. An additional evaluation where only negative examinations, followed by interval cancers that are diagnosed within a year, were included, is reported in Supplement B. Excluding cases with an interval cancer that is diagnosed later decreases the probability that true interval cancers are present in the group with assumed high masking risk. In the original work, only contralateral images were used to eliminate the influence of the lesion and allow Mammatus to only analyze the breast texture. An additional analysis using only mammograms of the contralateral breast was reported in Supplement C. A comparison of the calculated lesion masking risk of the contralateral and ipsilateral breast was reported in Supplement D.

The second cumulative ROC analysis [12] consisted of two discriminatory tasks among three types of cases: (1) mammograms with a screen-detected cancer that was visible as a mass (category 1, low masking risk), (2) mammograms before an interval cancer that had a visible mass in retrospect (category 3, intermediate masking risk), and (3) mammograms before an interval cancer without a visible mass in retrospect (category 4, high masking risk). Mammograms with a screen-detected cancer that was not visible as a mass (category 2) were excluded from this analysis. The AUC was used to compare Mammatus to VBD on both ROC analyses with a single-reader multi-case analysis (MRMCaov for R, version 0.3.0 [10, 11]) and a jackknife function to estimate performance metric covariances. A distribution of all AUC values was created by bootstrapping with 1,000 iterations, and a significance level of 0.05 was used for all *p*-values.

## Results

From the original set of 2610 diagnosed cancers, 1179 examinations met the inclusion criteria and were retrospectively interpreted with respect to lesion masking as shown in Fig. 1. For examinations with a screen-detected cancer, a mass was visible in at least one view in 82% (629/768) of the exams. For the examinations preceding interval cancers, a mass was visible in only 55% (228/411) of the examinations (*p* < 0.001). The lesion masking estimation algorithm was able to process 935 examinations successfully. Of the failed examinations, 4 were not processed correctly due to the presence of a breast implant, and 236 were due to a missing reference of the open-beam signal.

An overview of the patient and mammogram characteristics is presented in Table 1. Patient and mammogram characteristics of the original study are described in Supplement A. Examinations with a screen-detected cancer were acquired at a higher median age, 64 (IQR 59–70), than examinations before interval cancers (median: 58, IQR 53–65) (*p* < 0.001). Median VBD was higher in examinations preceding interval cancers, 8.3% (IQR 5.7–14.2) vs 5.9% (IQR 4.3–8.8) in examinations with screen-detected cancers (*p* < 0.001). The distribution of BIRADS density categories based on the VBD is shown in Table 1. There was no significant difference in the size of visible masses measured on mammograms with screen-detected vs interval cancers (*p* = 0.49). Interval cancers were diagnosed after a median of 13 (IQR 7–19) months, with no difference in time to diagnosis when the mass was visible at screening in retrospect or not (*p* = 0.18).

**Table 1:** Demographics of the study sample in the four categories of screen-detected cancers and screening examinations before interval cancers with both visible and non-visible masses

	Screen-detected cancer		Interval cancer	
	Visible mass	No visible mass	Visible mass	No visible mass
Nr examinations	527	108	165	139
Age at examination^*^ [years]	65 (59–70)	62 (57–68)	58 (53–65)	59 (54–66)
Breast thickness^×^ [mm]	62 (39–80)	59 (34–81)	60 (34–86)	58 (31–81)
Mean glandular dose* (mGy)	1.6 (1.4–1.9)	1.8 (1.6–2.1)	1.8 (1.5–2.0)	1.8 (1.4–2.1)
VBD*	5.5% (4.1–8.1)	9.1% (5.6–12.4)	8.0% (5.7–12.2)	9.3% (5.7–19.2)
BIRADS density				
A	184 (35%)	13 (12%)	19 (12%)	20 (14%)
B	192 (36%)	27 (25%)	56 (34%)	36 (26%)
C	128 (24%)	49 (45%)	66 (40%)	38 (27%)
D	22 (4%)	19 (18%)	24 (15%)	42 (30%)
Missing	1 (0%)	0	0	3 (2%)
Lesion size^*^ [mm^2^]	118 (70–196)		130 (66–204)	
Time to diagnosis^*^ [months]			12 (6–18)	15 (7–20)
Cancer type				
Ductal	404 (77%)	78 (72%)	102 (62%)	99 (71%)
Lobular	71 (13%)	24 (22%)	44 (27%)	27 (19%)
Other	50 (9%)	5 (5%)	16 (10%)	8 (6%)
Grade				
I	198 (38%)	36 (33%)	32 (19%)	28 (20%)
II	203 (39%)	48 (44%)	67 (41%)	51 (37%)
III	88 (17%)	16 (15%)	41 (25%)	38 (27%)
Undetermined	38 (7%)	8 (7%)	25 (15%)	22 (16%)
Mammatus score^*^	0.25 (0.17–0.34)	0.35 (0.28–0.46)	0.36 (0.25–0.44)	0.37 (0.27–0.49)

BIRADS density categories were obtained indirectly from the VBD estimation from Volpara

^*^ Median (interquartile range)

^×^ Mean (95% confidence interval)

### Comparison to the original study

**Fig. 2:**
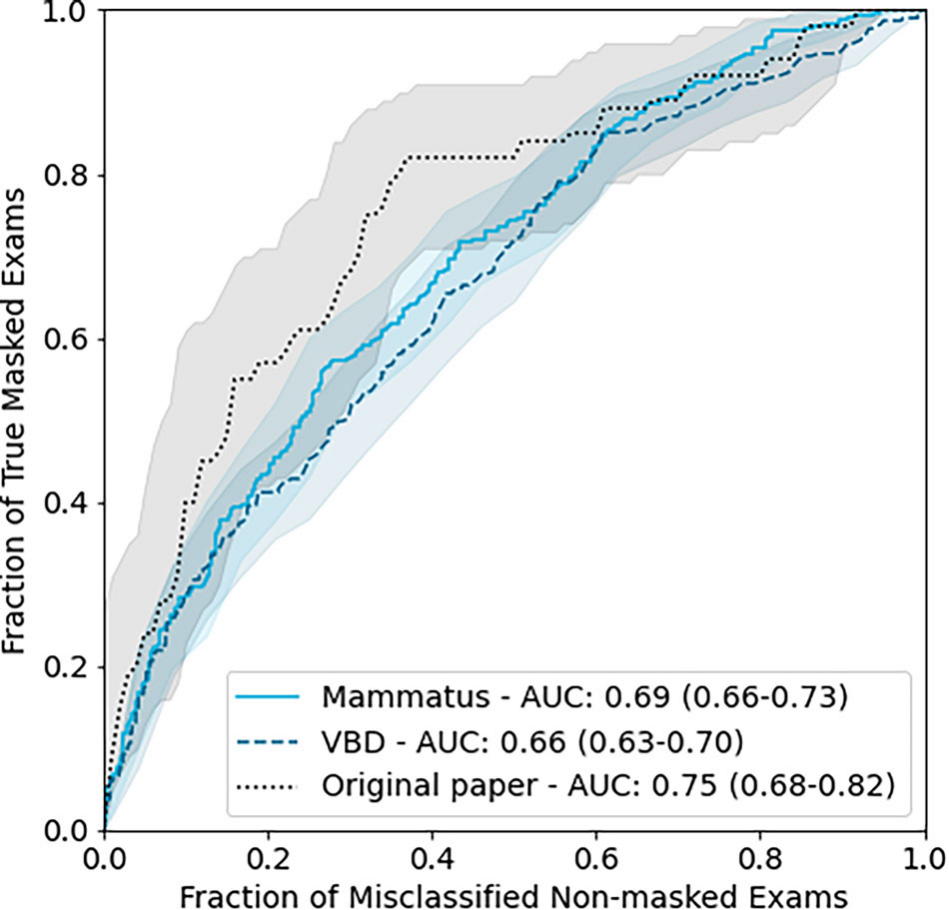
ROC curves of Mammatus and VBD to discriminate between examinations with a screen-detected cancer and before an interval cancer. For reference, the ROC curve of Mammatus from the original paper is also included [5]. The mean and 95% confidence intervals of the AUCs are included in the figure legend

Mammatus achieved an AUC of 0.69 (95% CI: 0.66–0.73) for discriminating between examinations with screen-detected cancers and negative examinations followed by interval cancers, as shown in Fig. 2 and Table 2. On this task, Mammatus outperformed VBD as a predictor, which achieved an AUC of 0.66 (95% CI: 0.63–0.70) (*p* = 0.019). The AUC of Mammatus was not significantly different from the AUC of 0.75 (0.68–0.82) reported in the original work by Mainprize et al [5] (*p* = 0.15). An evaluation where negative examinations followed by interval cancers that are diagnosed after a year were excluded is reported in Supplement B.

**Table 2:** Evaluation of Mammatus and VBD to discriminate between examinations with a screen-detected cancer and examinations before an interval cancer

	Screen-detected (low masking risk)	Interval cancers (high masking risk)	AUC
Mammatus	635	304	0.69 (0.66–0.73)
VBD	634	301	0.66 (0.63–0.70)
Original paper	147	67	0.75 (0.68–0.82)

For reference, the AUC of Mammatus from the original paper is also included [5]. The area under the receiver operating characteristic curve (AUC) is given as the mean and (95% confidence intervals)

### Three-category ROC

The results of the cumulative ROC analysis of Mammatus and VBD are shown in Table 3. Mammatus outperformed VBD on discriminating examinations with low vs intermediate and high masking risk (*p* = 0.019) but achieved similar results for discriminating examinations with low and intermediate vs high masking risk (*p* = 0.61).

**Table 3:** Cumulative ROC analysis of Mammatus and VBD to discriminate three categories; low masking risk (screen-detected with visible mass), intermediate masking risk (interval cancer with visible mass), and high masking risk (interval cancer without visible mass)

	Masking risk			AUC	
	Low	Intermediate	High	Low vs intermediate and high	Low and intermediate vs high
Mammatus	527	165	139	0.73 (0.70–0.76)	0.69 (0.64–0.74)
VBD	526	165	136	0.70 (0.67–0.73)	0.68 (0.63–0.73)

The area under the ROC curve (AUC) is given as the mean and (95% confidence intervals)

**Fig. 3:**
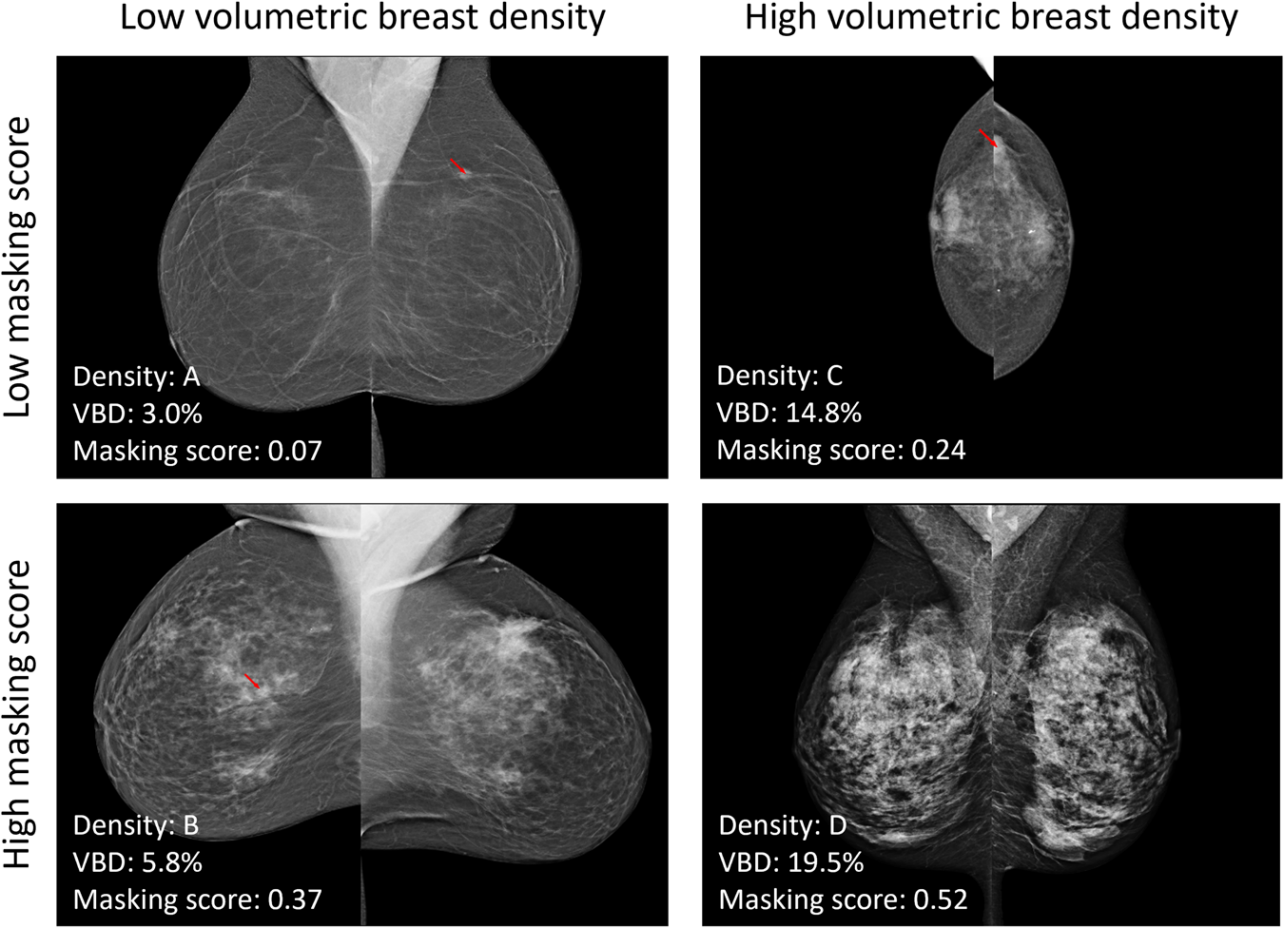
Examples of four examinations with low and high Mammatus masking scores and volumetric breast densities (VBD). If a mass was visible in retrospect, it is indicated with a red arrow. **A** Positive examination with a screen-detected cancer, age 64. **B** Negative examination scored as BI-RADS 1 before an interval cancer diagnosed 2 months later and visible in retrospect, age 53. **C** Negative examination scored as BI-RADS 2 before an interval cancer diagnosed after 6 months, age 72. **D** Negative examination scored as BI-RADS 1 before an interval cancer diagnosed after 6 months in the right breast, but not visible in retrospect, age 56

Four example examinations with low and high masking scores by Mammatus and low and high VBD are shown in Fig. 3. The first example shows an examination with a screen-detected lesion where both metrics are low. Here, the lesion is clearly visible, and the textures in the breast do not hinder the interpretation of the lesion. The second example shows an interval cancer that was visible in retrospect with low VBD but a high Mammatus score. From the mammogram, it is evident that the density is relatively low, but there are some clusters of fibroglandular tissue creating areas with a potential masking risk. Although the lesion is visible, it is partially obscured by the breast tissue. The third example again shows an interval cancer that was visible in retrospect. This examination had high VBD and a relatively low masking score. As can be expected, the combination of a high VBD and a low masking score is rare in this dataset. The last example shows a negative screening examination before an interval cancer was diagnosed six months later. It shows an extremely dense breast with a complex texture that could easily obscure a lesion, and this is reflected in its high VBD and Mammatus score.

## Discussion

The results of this study show that the performance of Mammatus on a large Dutch screening cohort is not statistically different from the original reported AUC on a North American cohort, even when including mammograms with present lesions. Furthermore, Mammatus outperforms VBD as a predictor of masking risk. A three-category ROC analysis based on retrospective mass visibility analysis showed that Mammatus is best suited to identify low masking risk while having a lower ability to discriminate between low plus intermediate vs high masking risk.

There are two main differences between the original cohort and this new cohort: differences in the population and differences in the image quality or characteristics. The cohort of the original paper used to develop the algorithm had a larger spread in ages and included women under 50, who are not eligible for the Dutch National Screening Program. Moreover, in the Netherlands, double reading of mammograms is the standard, while single reading is often used in North American screening. This can result in cancers being more easily missed in North American screening, and, therefore, some more mammographic examinations being classified as having high masking risk in the North American cohort that would not have been considered as such in the Dutch cohort. Another contributing factor is the difference in image characteristics between the cohorts. The mammograms in the original study were mainly acquired with GE mammography systems with images produced before 2010, while the Dutch National Screening Program uses only Hologic systems. Radiomic features, as used by Mammatus, can possibly be affected by differences in characteristics between images acquired by those systems [13, 14].

Few other studies have attempted to predict lesion masking risk in mammograms. One study used density maps and lesion probability maps to predict lesion masking [7]. However, results did not improve over lesion masking risk beyond that based on VBD alone. Another study used radiomics features of local image quality factor maps in addition to VBD [6]. On an internal set with screen-detected and interval cancers, VBD and their model achieved similar AUCs of 0.65 and 0.69, respectively. These results are similar to the results in this study, which were obtained on an external dataset. Other studies suggest that high breast thickness also increases masking risk, and breast thickness should be included when predicting lesion masking risk [4, 15].

The retrospective evaluation of the visibility of masses in negative examinations before an interval cancer allowed for a new category where lesions were initially missed but were visible in retrospect. In this study, 55% of interval cancers were visible as a mass in retrospect, including those showing minimal signs (as defined by the European Guidelines [16]). A Dutch study from a different screening region reported that 46% of all interval cancers were already visible in the last screening round, of which approximately half had minimal signs only [17]. Comparable results were found in Norway, which has a similar screening program, with 52% of all interval cancers visible, of which approximately half had only minimal signs [18].

The category of interval cancers that were visible in retrospect was labeled as having an intermediate masking risk. However, it could be argued that these lesions are not masked, because they are visible even though more challenging for radiologists. The results showed that Mammatus also assigns high masking scores to these cases, and, therefore, also partially captures the difficulty of reading the mammogram. Identifying these cases as needing to be re-evaluated by radiologists at screening could potentially reduce the interval cancer rate. Unlike interval cancers without a visible lesion, no other modality is necessary to detect these lesions, and it has been shown that re-evaluating mammograms in a batch with high prevalence can improve sensitivity [19]. The importance of finding these initially missed cancers is high since these cancers are often larger, have a more advanced tumor stage, and a higher proportion of mastectomy compared to true interval cancers [17]. True interval cancers are also included in the last category, where no lesion was visible in retrospect. Therefore, this category does not necessarily have a high lesion masking risk.

A model that can predict lesion masking risk could be used to identify cases that need to be re-evaluated or need to be offered screening by another modality. Before a model such as Mammatus can be deployed for this application, it needs to be tested on a normal screening population, where the majority of women are cancer-free, to ensure that not too many of these examinations are flagged as having high masking risk. This was already studied retrospectively in a North American cohort and is shown to be more efficient than using BI-RADS density [20]. The following research will include evaluating the potential benefit of Mammatus when using its predictions to optimize reading strategies in the Dutch National Breast Cancer Screening Program.

In conclusion, a previously developed lesion masking prediction model, Mammatus, outperformed VBD as a masking risk predictor, especially in distinguishing between mammograms with low risk of lesion masking vs intermediate/high risk. Mammatus showed no significant difference in performance compared to the original North American cohort. Such an algorithm could potentially be used to identify cases that need to be re-evaluated to find an initially missed cancer or those who need to be offered screening by another modality to reduce the risk that a cancer is missed due to masking.

## Supplementary information

ELECTRONIC SUPPLEMENTARY MATERIAL

## Abbreviations

AUC: Area under the receiver operator characteristic curve
ROC: Receiver operator characteristics
VBD: Volumetric breast density
