# Supplementary material for: More than density: validating a mammographic masking prediction model in Dutch breast cancer screening
Source: Eur Radiol. 2025 May 29;35(12):8191–9. doi: 10.1007/s00330-025-11687-x (PMC12634747; doi:10.1007/s00330-025-11687-x)
Supplement: Supplementary file 1 — ELECTRONIC SUPPLEMENTARY MATERIAL [file 330_2025_11687_MOESM1_ESM.pdf]

More than Density: Validating a Mammographic Masking Prediction Model in Dutch Breast Cancer Screening

ELECTRONIC SUPPLEMENTARY MATERIAL

A - Descriptive table of original paper

Table S1. Demographics of the study sample from the original work by Mainprize et al[5] for both screen-detected cancers and screening examinations before interval cancers. Values are obtained from Tables 2 and 3 from the original work[5].

	Screen-detected cancer	Interval cancer
**Nr examinations**	147	67
**Age at examination* [years]**	64 (56-72)	57 (48-64)
**Breast volume* [cm3]**	915 (559-1128)	674 (362-818)
**VBD (Volpara) [%]**	8.0 (4.1-9.6)	11.9 (5.8-16.6)
**VBD (in-house) [%]**	14.0 (9.0-15.6)	20.7 (11.9-27.8)
**BIRADS density**		
**A**	30 (20.4%)	2 (3.0%)
**B**	62 (42.2%)	27 (40.3%)
**C**	46 (31.3%)	30 (44.8%)
**D**	9 (6.1%)	8 (11.9%)
**BMI**		
**< 22.8**	30 (20.4%)	22 (32.8%)
**22.8 – 26.4**	38 (25.9%)	16 (23.9%)
**26.4 – 30.6**	37 (25.2%)	17 (25.4%)
**> 30.6**	42 (28.6%)	12 (17.9%)
**Race**		
**White**	128 (87.1%)	52 (77.6%)
**Black**	16 (10.9%)	14 (20.9%)
**Other**	1 (0.7%)	0 (0.0%)
**Unknown**	2 (1.4%)	1 (1.5%)
**Family history of breast cancer**		
**None**	77 (52.4%)	31 (46.3%)
**First degree relative**	36 (24.5%)	23 (34.3%)
**Second degree relative**	34 (23.1%)	13 (19.4%)
**Year of examination**		
**2003 – 2005**	31 (21.1%)	26 (38.8%)
**2006 – 2008**	48 (32.7%)	17 (25.4%)
**2009 – 2011**	48 (31.3%)	20 (29.9%)
**2012 – 2013**	22 (15.0%)	4 (6.0%)
**Method of detection**		
**Mammogram**	147 (100%)	0 (0.0%)
**Other imaging**	0 (0.0%)	7 (10.4%)
**Clinical[x]**	0 (0.0%)	60 (89.6%)

*median (interquartile range), [x]A clinical method of detection refers to a palpable lump or other breast symptoms (pain or nipple discharge).

B - Interval cancers diagnosed within 1-year

Mammatus achieved an AUC of 0.75 (95% CI 0.70-0.79) for discriminating between examinations with screen-detected cancers and negative examinations followed by interval cancers that are diagnosed within a year, as shown Table S2. On this task, Mammatus outperformed VBD as a predictor, which achieved an AUC of 0.71 (95% CI 0.66-0.76) (p = 0.017). In the original work by Mainprize et al[5], a similar analysis was done with interval cancer diagnosed within a year. The only difference being that only screen-detected cancers were included that were preceded by a negative mammogram maximally 13 months ago. The AUC of this analysis did not significantly differ from the AUC of Mammatus in the Dutch screening cohort (p = 0.44).

Table S2. Evaluation of Mammatus and volumetric breast density (VBD) to discriminate between examinations with a screen-detected cancer and examinations before an interval cancer that was diagnosed within 12 months. For reference, the AUC curve of Mammatus from the original paper is also included[5]. The area under the receiver operating characteristic curve (AUC) is given as mean and (95% confidence intervals).

	**Screen-detected** *(Low masking risk)*	**Interval cancers** *(High masking risk)*	**AUC**
**Mammatus**	635	145	0.75 (0.70-0.79)
**VBD**	634	144	0.71 (0.66-0.76)
**Original paper**	70	44	0.79 (0.69-0.87)

C Contralateral analysis

In the original paper[5], only mammograms of the contralateral breast were analyzed to eliminate the influence of the lesion and allow Mammatus to only analyze the breast texture. The breast texture is assumed to be similar between both breasts. In the Dutch cohort, Mammatus was applied to the mammograms of both breasts. This supplement provides an analysis where Mammatus was only applied to mammograms of the contralateral breast as well as the volumetric breast density (VBD) only calculated on the contralateral breast.

When only mammograms of the contralateral breast were used, Mammatus achieved an AUC of 0.70 (95% CI 0.67-0.74) for discriminating between examinations with screen-detected cancers and negative examinations followed by interval cancers, as shown in Figure S1 and Table S3. On this task, Mammatus outperformed VBD as a predictor, which achieved an AUC of 0.66 (95% CI 0.63-0.70) ($p = 0.0018$). The AUC of Mammatus was not significantly different from the AUC of 0.75 (0.68-0.82) reported in the original work by Mainprize et al[5] ($p = 0.22$).

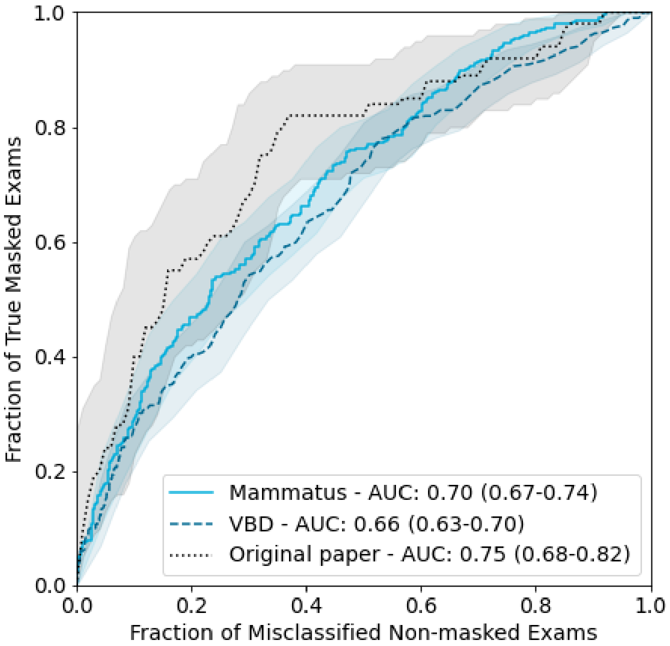

Figure S1. Receiver operating characteristics (ROC) curves of Mammatus and volumetric breast density (VBD) to discriminate between contralateral examinations with a screen-detected cancer and before an interval cancer. For reference, the ROC curve of Mammatus from the original paper is also included[5]. The mean and 95% confidence intervals of the AUCs are included in the figure legend.

Table S3. Evaluation of Mammatus and volumetric breast density (VBD) to discriminate between examinations with a screen-detected cancer and examinations before an interval cancer evaluated only on the contralateral breast. For reference, the AUC curve of Mammatus from the original paper is also included[5]. The area under the receiver operating characteristic curve (AUC) is given as mean and (95% confidence intervals).

	**Screen-detected** *(Low masking risk)*	**Interval cancers** *(High masking risk)*	**AUC**
**Mammatus**	632	303	0.70 (0.67-0.74)
**VBD**	628	299	0.66 (0.63-0.70)
**Original paper**	147	67	0.75 (0.68-0.82)

D Ipsilateral versus contralateral mammograms

The lesion masking score of Mammatus was 0.012 ± 0.053 (mean ± std) ($p<0.001$) higher for ipsilateral mammograms compared to contralateral mammogram (Table S4). Figure S2 shows the Bland-Altman plots of the lesion masking score for the different ground truth groups. This subgroup analysis shows that the lesion masking score was significantly higher for the ipsilateral breast of screen detected cancers, but not for interval cancers, regardless of the presence of a visible mass.

VBD was 0.2% ± 2.3% (mean ± std) ($p=0.002$) higher for ipsilateral mammograms compared to contralateral mammogram (Table S4). However, the subgroup analysis shows that this difference is only present for cases with a screen detected cancer with a visible mass (Figure S3). Moreover, the differences in VBD are small compared to the VBD range found in this population.

Table S4. Difference in lesion masking score and VBD between mammograms of the contralateral and ipsilateral breast. Values are given in mean ± standard deviation, with the corresponding p-value calculated with a 1-sample t-test.

	**Mammatus lesion masking score**		**Volumetric breast density**	
**All examinations**	0.012 ± 0.053	<0.001	0.2 ± 2.3	0.002
**Screen detected cancers**				
**Visible mass**	0.018 ± 0.055	<0.001	0.2 ± 1.6	0.020
**No visible mass**	0.015 ± 0.043	<0.001	0.4 ± 2.7	0.12
**Interval cancers**				
**Visible mass**	0.002 ± 0.050	0.65	0.4 ± 2.8	0.078
**No visible mass**	0.001 ± 0.052	0.89	0.2 ± 3.6	0.46

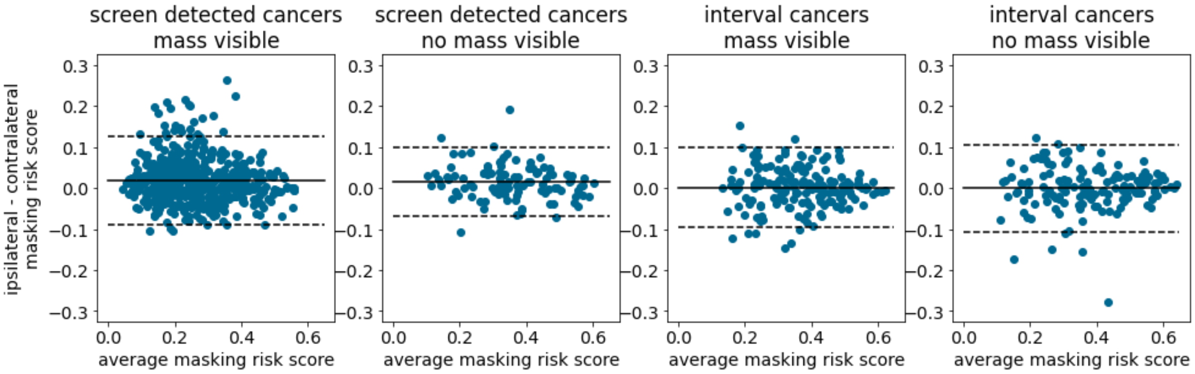

Figure S2. Bland-Altman plots comparing the lesion masking risk calculated on the mammograms of the ipsilateral and contralateral breast, separated on ground truth category. The solid line indicates the average difference, the dashed lines indicate the 95% confidence interval at ± 1.96 standard deviation.

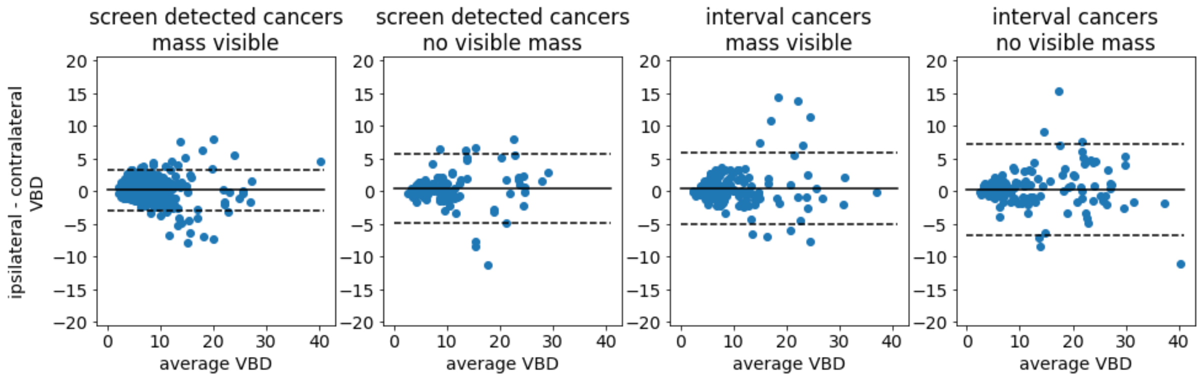

Figure S3. Bland-Altman plots comparing the volumetric breast density (VBD) calculated on the mammograms of the ipsilateral and contralateral breast, separated on ground truth category. The solid line indicates the average difference, the dashed lines indicate the 95% confidence interval at ± 1.96 standard deviation.
